# Dermoscopy as an auxiliary tool for the diagnosis of acral squamous diseases: palmoplantar psoriasis, tinea pedis/manuum and eczema^⋆^

**DOI:** 10.1016/j.abd.2023.05.008

**Published:** 2024-04-23

**Authors:** Mariana Vieira Martins Sampaio Drummond, Jules Rimet Borges, Ana Maria Quinteiro Ribeiro, Bárbara Alvares Salum Ximenes

**Affiliations:** Service of Dermatology, Hospital das Clínicas de Goiânia, Goiânia, GO, Brazil

Dear Editor,

Dermoscopy is an imaging exam that allows the observation of structures located in the skin, not seen with the naked eye. The equipment called a dermatoscope, makes it possible to see high-resolution images by reducing surface reflectance.^1^

In recent years, dermoscopy has become an important auxiliary tool for the non-invasive diagnosis of several dermatological diseases, including scalp and hair diseases (trichoscopy), nail and nail fold abnormalities (onychoscopy), skin infections and infestations (entomodermoscopy) and inflammatory dermatoses (inflammatoscopy).^2^

One of the inflammatory diseases evaluated through dermoscopy is psoriasis, a chronic, immune-mediated disease that affects approximately 125 million people worldwide, with the palmoplantar variant accounting for 11% to 39% of these patients. It is divided into two forms: hyperkeratotic (thick scaly plaques) and pustular.^3^

Another inflammatory dermatosis where diagnosis can be aided by dermoscopy is acral eczema, a chronic disease with a significant negative impact on patient quality of life and associated with an economic burden on society due to the impairment of the individual working capacity. Its average annual prevalence is 10%, with a higher incidence in women, up to 30 years of age.^4^

Tinea manuum and tinea pedis can be best evaluated through dermoscopy. Tinea is caused by dermatophyte fungi that invade the keratinized layers of the skin and its appendages, causing dermatophytoses. These affect 20% to 25% of the world's population and are the most common fungal infections worldwide.^5^

Psoriasis, dermatophytosis, and acral eczema are three different dermatological conditions, with similar clinical manifestations and high prevalence nationally and worldwide. Scientific investment aimed at helping to differentiate them, such as dermoscopy, will positively affect patients, professionals, and the health system itself.

This is an analytical, cross-sectional study of diagnostic accuracy. After approval by the Research Ethics Committee according to CAAE number 52926621.7.0000.5078, data collection was carried out at the Dermatology Service of Hospital das Clínicas de Goiânia, from April 20, 2021 to April 29, 2022. The selected sample was a consecutive one, consisting of patients with scaly palms or soles.

The inclusion criteria were: confirmed diagnosis of palmoplantar psoriasis, acral eczema, tinea manuum or tinea pedis; outpatient consultation at the dermatology service in Hospital das Clínicas de Goiânia; signing the TCLE (Free and Informed Consent Form); age over 18 years old. Patients with a clinical diagnosis of psoriasis vulgaris with acral involvement or palmoplantar psoriasis confirmed by pathological examination were included; clinical diagnosis of contact/atopic eczema with acral involvement confirmed by pathological examination; or diagnosis of tinea manuum or pedis confirmed by direct mycological examination for fungi.

The analysis of the photographs was carried out by two dermatologists with experience in dermoscopy, who filled out the table without knowledge of the patient diagnosis. Data were analyzed and organized in a qualitative and quantitative way, through the identification of “n”, sensitivity and specificity.

Data were collected from 45 patients with scaling on the palmar or plantar region. Of these, 33 patients met the inclusion criteria, 13 with eczema, 12 with psoriasis and 8 with tinea.

Table 1 shows the frequency of dermoscopic findings in each diagnosis. Table 2 shows data regarding the sensitivity and specificity of the dermoscopic signs mentioned before in relation to the studied diseases.

**Table 1:** Frequency of dermoscopic findings in each diagnosis.

	Eczema (n = 13)		Psoriasis (n = 12)		*Tinea* (n = 8)	
	n	%	n	%	n	%
**Punctate vessels**						
Focal	1	7.7	2	16.7	0	0.0
Diffuse	1	7.7	5	41.7	1	12.5

**White scales**						
In creases	1	7.7	0	0.0	8	100.0
Diffuse	6	46.2	4	33.3	0	0.0

**White and yellow scales**	6	46.2	8	66.7	1	12.5

**Brownish dots and/or globules**	2	15.4	5	41.7	0	0.0

**Table 2:** Sensitivity (S) and specificity (Sp) of dermoscopic signs for the diagnosis of eczema, psoriasis and tinea.

	Eczema	Eczema	Psoriasis	Psoriasis	Tinea	Tinea
Dermoscopic signs	S	Sp	S	Sp	S	Sp
Focal punctate vessels	**7.7**	**90.0**	16.7	95.2	0.0	88.0
Diffuse punctate vessels	7.7	70.0	**41.7**	**90.5**	12.5	76.0
White scales in skin creases	7.7	60.0	0.0	57.1	**100.0**	**96.0**
Diffuse white scales	46.2	80.0	**33.3**	**71.4**	0.0	60.0
White and yellow scales	**46.2**	**55.0**	66.7	66.7	12.5	44.0
Brownish dots and/or globules	**15.4**	**75.0**	41.7	90.5	0.0	72.0

For eczema, the most common dermoscopic findings were diffuse white scales and white and yellow scales. Sensitivity was higher for white and yellow scales (46.2%). Specificity was 90% for focal punctate vessels. In psoriasis, the most frequent finding was white and yellow scales, present in 2/3 of patients. The most sensitive and specific dermoscopic findings for the diagnosis of tinea pedis/manuum were white scales in skin creases.

The dermoscopic findings sought in each disease are demonstrated as follows. Fig. 1 shows white scales in skin creases in a patient with tinea pedis; Fig. 2 shows punctate vessels and diffuse white scales in a patient with psoriasis and Fig. 3 shows focal punctate vessels, white and yellow scales, and brownish globules in a patient with palmar eczema.

**Figure 1:**
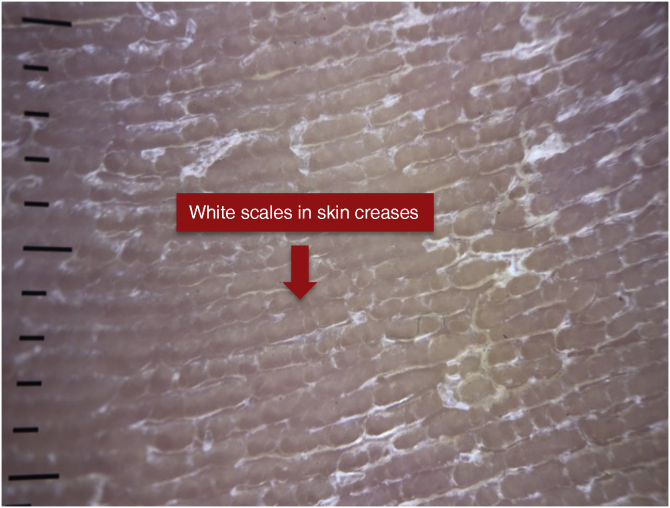
Dermoscopy of a patient with tinea pedis.

**Figure 2:**
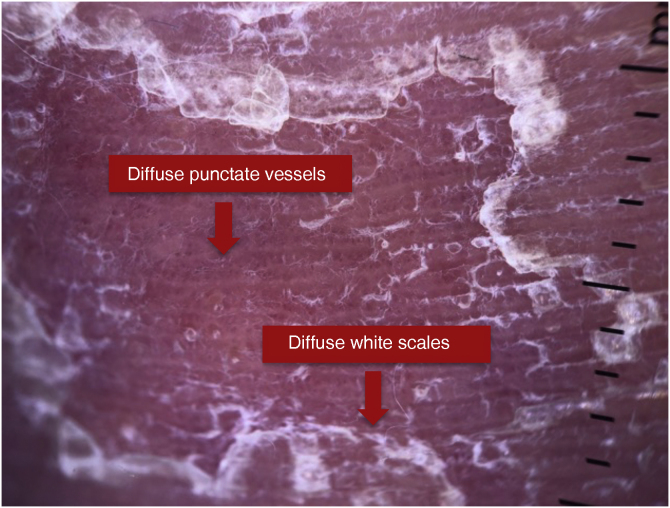
Dermoscopy of a patient with psoriasis.

**Figure 3:**
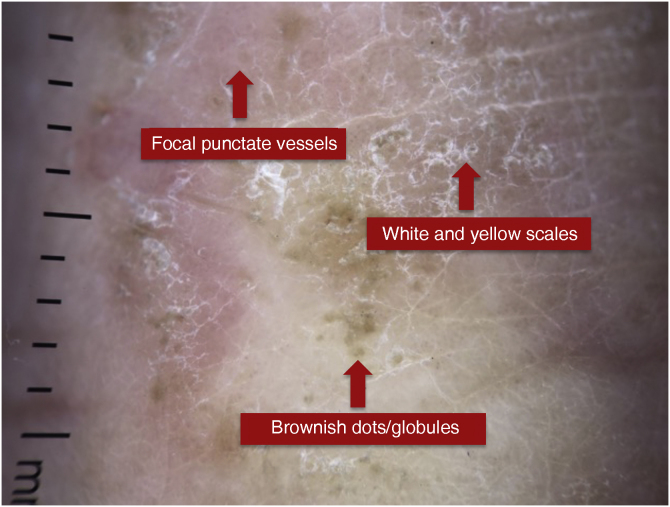
Dermoscopy of a patient with palmar eczema.

Dermoscopy has become important in areas besides dermato-oncology for the early diagnosis of melanoma and non-melanoma skin cancers. Its applicability has expanded to the definition of dermoscopic patterns for several other dermatoses, including inflammatory ones. Moreover, dermoscopy is not only used for diagnostic purposes but also to evaluate therapeutic progress and response.^6^

A recent literature review demonstrated that in plaque psoriasis, dermoscopy reveals the presence of regularly distributed punctate vessels with a light red background and diffuse white scales, with 88% specificity and 84.9% sensitivity.^6^ In the present study, the findings of diffuse punctate vessels showed 90.5% specificity and 41.7% sensitivity in patients with palmoplantar psoriasis. Diffuse white scales showed 71.4% specificity and 33.3% sensitivity, strongly supporting the diagnosis of psoriasis, but their absence does not rule out the diagnosis.

In the 2016 study by Errichetti and Stinco, the most common dermoscopic findings in palmar psoriasis were white scales on an evident erythematous background (all patients, 100%) with a diffuse distribution in 80% of them. Furthermore, regularly distributed punctate vessels were also observed in four patients (40%).^7^ In comparison in the current study, diffuse punctate vessels were observed in 41.7% and diffuse white scales in 33.3% of patients with psoriasis.

Eczema or “dermatitis” comprise several clinical entities, such as contact/allergic dermatitis, atopic dermatitis, and seborrheic dermatitis that share characteristic histopathological changes.^6^, ^8^ Regarding palmoplantar eczema, the most frequent dermoscopic findings are yellowish desquamation with or without white scales, yellowish crusts, and focal punctate vessels. The brownish-orange dots/globules correspond to spongiotic vesicles not visible to the naked eye.^7^ Errichetti and Stinco in 2016 showed that in 11 patients with chronic hand eczema, 90.9% had yellowish scales distributed focally and 72.7% had brownish-orange dots/globules on an erythematous background.^7^ In the present study, of 13 patients with palmar or plantar eczema, only 46.2% had white or yellow scales, and 15.4% had brownish dots or globules.

The same authors mentioned above observed that diffuse white scales more strongly suggest the diagnosis of palmoplantar psoriasis, while the detection of yellowish scales, brownish-orange dots/globules, and yellowish-orange crusts support the diagnosis of eczema. The color of the scales (white *versus* yellow) is extremely useful information to aid in the differential diagnosis of acral squamous diseases.^7^, ^9^ However, the present study showed that in patients with psoriasis, only 33.3% of patients had white scales only, while 66.7% had white and yellow scales. On the other hand, in patients with eczema, the same percentage was observed for those with white and yellow scales and those with white scales only, which was 46.2%.

Regarding the dermoscopic findings of tinea manuum/pedis, the same authors observed whitish scales located mainly in skin creases, a finding that is absent in palmar psoriasis and chronic eczema of the hands.^10^ In the present study, 100% of patients with tinea manuum/pedis had white scales in skin creases, with 100% sensitivity and 96% specificity. White scales do not differentiate it from other scaly diseases; however, its typical location in the creases, a predilection site for dermatophytes, helps in differentiating it from other diseases.^10^

As a limitation of this study, it is important to highlight that the dermoscopy photos of a part of the lesion does not represent its entirety. Therefore, some dermoscopic findings may not have been recorded at the time of the photograph but were still present in the lesion. Additionally, the time between the anatomopathological diagnosis of patients with psoriasis and eczema and the application of dermoscopy was not evaluated, as well as whether or not the patient was undergoing treatment at the time of the evaluation. It is concluded that, despite dermoscopic characteristics suggesting a certain disease, they are not specific since, as observed in this study, some characteristics observed more frequently in patients with acral eczema were also present in patients with palmoplantar psoriasis. Therefore, clinical correlation and adequate anamnesis are extremely important, in addition to dermoscopy.

It is noteworthy that 100% of patients with tinea mannum/pedis had scales in skin creases, with a sensitivity of 100% and specificity of 96%, which is a very suggestive finding for this diagnosis and highlights the role of dermoscopy in the diagnosis of this disease.

## Financial support

None declared.

## Authors' contributions

Mariana Vieira Martins Sampaio Drummond: Design and planning of the study; collection, analysis and interpretation of data; statistical analysis; drafting the manuscript or revising it critically for important intellectual content; collection, analysis and interpretation of data; intellectual participation in the propaedeutic and/or therapeutic conduct of the studied cases; critical review of the literature.

Jules Rimet Borges: Design and planning of the study; drafting the manuscript or revising it critically for important intellectual content; collection, analysis and interpretation of data; effective participation in research orientation; intellectual participation in the propaedeutic and/or therapeutic conduct of the studied cases; critical review of the literature; approval of the final version of the manuscript.

Ana Maria Quinteiro Ribeiro: Data collection, or analysis and interpretation of data; statistical analysis; drafting the manuscript or revising it critically for important intellectual content; effective participation in research orientation.

Bárbara Alvares Salum Ximenes: Drafting of the manuscript or critical review of important intellectual content.

## Conflicts of interest

None declared.
